# A Novel Case of Fibroids after Menopause

**DOI:** 10.1155/2018/9104719

**Published:** 2018-12-25

**Authors:** Jade Edwards, Hamza Katali

**Affiliations:** ^1^ST5 Trainee in Obstetrics and Gynaecology, West Midlands Deanery, Birmingham, UK; ^2^Consultant Obstetrician and Gynaecologist at Hereford County Hospital, Stonebow Road, Hereford, UK

## Abstract

Fibroids are a condition commonly seen as part of routine gynaecological practice and generally present with menorrhagia, infertility, or pressure symptoms depending on their exact location. We report the case of a postmenopausal 57-year-old lady presenting with left-sided pain and frequency with a complex 15 cm mass mid pelvis and an adjacent additional 7 cm cyst but a risk of malignancy index suggesting benign pathology. She underwent total abdominal hysterectomy (TAH) and right-sided salpingo-oophrectomy (RSO) on recommendation of the multidisciplinary team (MDT) and histology confirmed leiomyomatosis peritonealis disseminata. This rare but important condition is often confused with peritoneal carcinomatosis on imaging. The mainstay of treatment involves lesion and omental excision followed by removal of hormonal stimuli although this approach must be personalised as many younger women may wish to retain their uteri. Long-term follow-up is essential due to the potential for malignant transformation.

## 1. Introduction

Fibroids are one of the commonest benign gynaecological conditions, occurring in almost 1 in 3 women over the age of 30 [1]. Benign tumours of the myometrial smooth muscle are seen more commonly in women of Afro-Caribbean descent and, as noted by Sarris et al. [1], vary enormously in size, location, and presenting symptoms. Fibroids impacting on the uterine cavity may cause menorrhagia or infertility whilst larger tumours filling the abdomen will distend the abdomen and put pressure on other organs [1]. We report a case of a patient who presented with multiple pelvic tumours, subsequently established to be multiple disseminated fibroids.

## 2. Case

A 57-year-old postmenopausal lady, para 0 with an unremarkable smear history, presented to the gynaecology department with a 3-month history of left-sided abdominal pain and frequency of micturition. She previously had a laparoscopic left-sided salpingo-oophrectomy for a benign mucinous cystoadenoma 3 years earlier. Clinical examination confirmed a pelvic mass arising from the pelvis. There were no features suggestive of an acute abdomen. An ultrasound scan demonstrated a large complex thick-walled cyst mid pelvis measuring 15 × 13 × 12 cm displacing the uterus to the right. There was an additional 7 × 6 cm complex cyst seen adjacent to this mass. Neither ovary was subsequently identified. The Ca125 was 8, giving a risk of malignancy index (RMI) of 24. A subsequent MRI pelvis was consistent with right ovarian cystic adenoma/cyst adenocarcinoma, and a bulky postmenopausal fibroid uterus containing multiple fibroids displaced to the right of the midline. The patient was referred to the MDT and total abdominal hysterectomy, right salpingo-oophrectomy, and omental biopsy were recommended in view of the potential for malignant diagnosis. This was completed uneventfully. At operation, the findings confirmed a multi-fibroid uterus with a large right-sided cystic mass. She made a good recovery postoperatively and a follow-up CT undertaken 6 months after surgery did not show any evidence of disease recurrence. She was commenced on letrozole 2.5mg daily in view of the histology results. Oestrogen results were not measured pre- or postoperatively.

## 3. Histology

Histology confirmed leiomyomatosis peritonealis disseminata–oestrogen receptor positive but no obvious malignant cells.

## 4. Discussion

Psathas et al. [2] define leiomyomatosis peritonealis disseminata (LPD) as a rare disorder characterised by the abnormal proliferation of smooth muscle containing nodules on peritoneal and subperitoneal surfaces. There are approximately 100 cases described in the literature worldwide although it is difficult to ascertain the true prevalence as many women remain asymptomatic and subsequently do not seek gynaecological assessment [2]. Covarrubias et al. [3] note it is seen generally in young women but cases after the menopause and even in male patients have been described. Symptoms, if seen at all, are usually nonspecific and include early satiety and abdominal distension [3].

The pathogenesis is currently uncertain, although multiple theories have been postulated by Yang et al. (2009) including peritoneal metaplasia, alterations in oestrogen and progesterone levels, genetics or iatrogenic factors, and most notably the dissemination of fragments of tissue through the peritoneal cavity secondary to fibroid morcellation at laparoscopic myomectomy.

Its appearance on imaging may be most commonly confused with peritoneal carcinomatosis, although other primary peritoneal malignancies, liposarcoma, lymphoproliferation, and disseminated endometriosis are also amongst the differentials [3]. Yang et al. identified in their 2015 paper that there are no existing reliable diagnostic tests; diagnosis is usually confirmed on histology or intraoperative findings. Histopathology will show smooth muscle fascicles with or without mitotic figures in interdigital fascicles [2]. In contrast to a leiomyosarcoma, there is no evidence of cell necrosis or atypia, hyperchromasia, or nuclear polymorphism [2].

Psathas et al. [2] propose that, in keeping with such a rare condition, there is no standard treatment rationale for cases of LPD. As most cases occur in women of reproductive age who are yet to complete their families, uterine conservation is a key concern [4]. The mainstay of treatment in these women generally involves lesion excision and omentectomy followed by removal of any hormonal stimuli [4]. Yang et al. [4] suggest that this could involve cessation of hormonal contraception or termination of pregnancy followed by administration of GnRH analogues, oestrogen receptor antagonists, or aromatase inhibitors. For those women who have completed their family, total abdominal hysterectomy, bilateral salpingo-oophrectomy, debulking, and omentectomy offers a more definitive alternative [4].

Although the majority of cases of LPD are benign, there remains potential for malignant transformation and, in view of the young age of many of these patients, long-term follow-up is essential [4]. Debulking surgery may need to be performed for recurrent disease. The use of systemic chemotherapy for nonresectable lesions or metastatic disease has also been described [5].

## 5. Conclusion

LPD remains a rare but important differential for disseminated peritoneal carcinomatosis, particularly when seen in women of reproductive age. Although conservative management may be offered after surgery, long-term surveillance is essential to reduce the impact of progressive disease or malignant transformation.
